# Relationship between intervertebral disc height and post operative dysphagia secondary to single-level anterior cervical discectomy and fusion- a retrospective study

**DOI:** 10.1186/s12891-024-07461-7

**Published:** 2024-05-10

**Authors:** Beiduo Shen, Zhiqiang Gao, Bijun Wang, Yufeng Huang, Desheng Wu

**Affiliations:** 1 Department of Spine Surgery, Shanghai East Hospital, School of Medicine, Tongji University, Shanghai, 200092 China; 2 Department of Bone & Joint Surgery, Shanghai East Hospital, School of Medicine, Tongji University, Shanghai, 200092 China

**Keywords:** Dysphagia, ACDF, Intervertebral disc height, C2-7 angle, Spinous process distance

## Abstract

**Background:**

One goal of Anterior Cervical Discectomy and Fusion (ACDF) is to restore the loss of intervertebral disc height (IDH) results from the degenerative process. However, the effects of IDH on postoperative dysphagia after ACDF remain unclear.

**Methods:**

Based on the results of a one-year telephone follow-up, A total of 217 consecutive patients after single-level ACDF were enrolled. They were divided into dysphagia and non-dysphagia groups. The age, BMI, operation time and blood loss of all patients were collected from the medical record system and compared between patients with and without dysphagia. Radiologically, IDH, spinous process distance (SP) of the operated segment, and C2-7 angle (C2-7 A) were measured preoperatively and postoperatively. The relationship between changes in these radiological parameters and the development of dysphagia was analyzed.

**Results:**

Sixty-three (29%) cases exhibited postoperative dysphagia. The mean changes in IDH, SP, and C2-7 A were 2.84 mm, -1.54 mm, and 4.82 degrees, respectively. Changes in IDH (*P* = 0.001) and changes in C2-7 A (*P* = 0.000) showed significant differences between dysphagia and non-dysphagia patients. Increased IDH and increased C2-7 A (*P* = 0.037 and 0.003, respectively) significantly and independently influenced the incidence of postoperative dysphagia. When the change in IDH was ≥ 3 mm, the chance of developing postoperative dysphagia for this patient was significantly greater. No significant relationship was observed between the change in spinous process distance (SP) and the incidence of dysphagia. The age, BMI, operation time and blood loss did not significantly influence the incidence of postoperative dysphagia.

**Conclusion:**

The change in IDH could be regarded as a predictive factor for postoperative dysphagia after single-level ACDF.

## Introduction

ACDF is one of the most common and effective surgical treatments for various cervical spine pathologies and is considered the gold standard treatment for cervical radiculopathy or myelopathy [1, 2]. According to the National Inpatient Sample (NIS) database, 1,059,403 ACDF procedures were performed in America from 2006 to 2013 [3]. ACDF is preferred by spine surgeons due to its generally excellent outcomes, shorter average hospital stay (< 2 days), lower incidence of postoperative mortality (0%), lower unplanned readmissions (4.4%), and lower overall complications (3.9%) [4]. Dysphagia is the most common complaint for patients after ACDF, ranging in incidence from 1.7 − 67% [5]. Although most cases are self-limiting, some patients suffer intolerable dysphagia, even remaining a lifetime [5, 6].

Two goals of ACDF are to restore the loss of middle-lower cervical curvature and height resulting from the degenerative process. Recent studies extensively investigated the impact of middle-lower cervical curvature changes on dysphagia after ACDF, with published data showing that the change of C2-7 A can serve as an effective predictive factor for dysphagia after ACDF [7–9]. It was found that the change of C2-7 A was the only statistically significant predictor of dysphagia at 2 and 6 weeks postoperatively after ACDF [7]. Additionally, the change of C2-7 A may play an important role in the development of dysphagia in both anterior and posterior cervical surgery [8], and when C2-7 A change was greater than 5°, the chance of developing postoperative dysphagia was significantly greater [9].

However, few studies have correlated postoperative dysphagia after ACDF with changes in middle-lower cervical height, such as IDH and SP. Recently, a large-scale retrospective radiologic analysis showed that height loss was independently associated with middle-lower cervical lordosis [10]. Moreover, it has been reported that the mean increase in IDH after ACDF was 2.62 mm [11], and distracting the disc space 3.0 mm beyond its original height led to significant biomechanical changes [12]. In a study of biomechanics of cervical spondylosis, IDH loss caused ventral angulation and eventual loss of cervical lordosis during cervical degeneration [13]. Therefore, the primary hypothesis is that changes in middle-lower cervical height after single-level ACDF, such as IDH and SP, may cause postoperative dysphagia after single-level ACDF.

## Materials and methods

### Patients Selection

Between June 2016 and October 2019, 217 consecutive patients (96 males and 121 females) underwent single-level ACDF at Shanghai East Hospital were included in this study. All patients were followed for at least 1 year. All patients in this study were performed by our team, and all patients received identical preoperative and postoperative management.

Exclusion criteria included: (1) abnormal swallowing function preoperatively; (2) upper respiratory or digestive tract disease; (3) previous cervical spine surgery; (4) cases not involving the cervical plating system; (5) cervical plain radiographs were not qualified.

### Surgical Protocols

All ACDF procedures were conducted via the anterior Smith-Robinson approach. The plating system used included the Vectra titanium plate and Cervios Peek interbody fusion device from Johnson & Johnson.

### Evaluation of Dysphagia

The presence, duration, and degree of postoperative dysphagia were recorded through telephone questionnaire. During the interview, four questions based on the dysphagia classification scale created by Bazaz et al(14) were asked. Question 1: Did you experience any swallowing difficulty immediately after ACDF? Question 2: How long did the swallowing difficulty last? Question 3: Did you have swallowing difficulty when drinking water or porridge? Question 4: Did you experience swallowing difficulty when eating dry or hard foods? If the answer to question 1 was “None,” these patients were defined as having “no dysphagia.” Patients who answered “Rare” to question 4 were defined as having “Mild dysphagia.” “Moderate dysphagia” was defined as occasional dysphagia with dry or hard food, and “Severe dysphagia” was defined as frequent swallowing difficulties with water or porridge.

### Data Collection

Demographic data included gender, age, BMI, blood loss, operation time were collected during the review of patients’ information in the clinical record system (Table 1).

### Radiographic Measurements

**Fig. 1:**
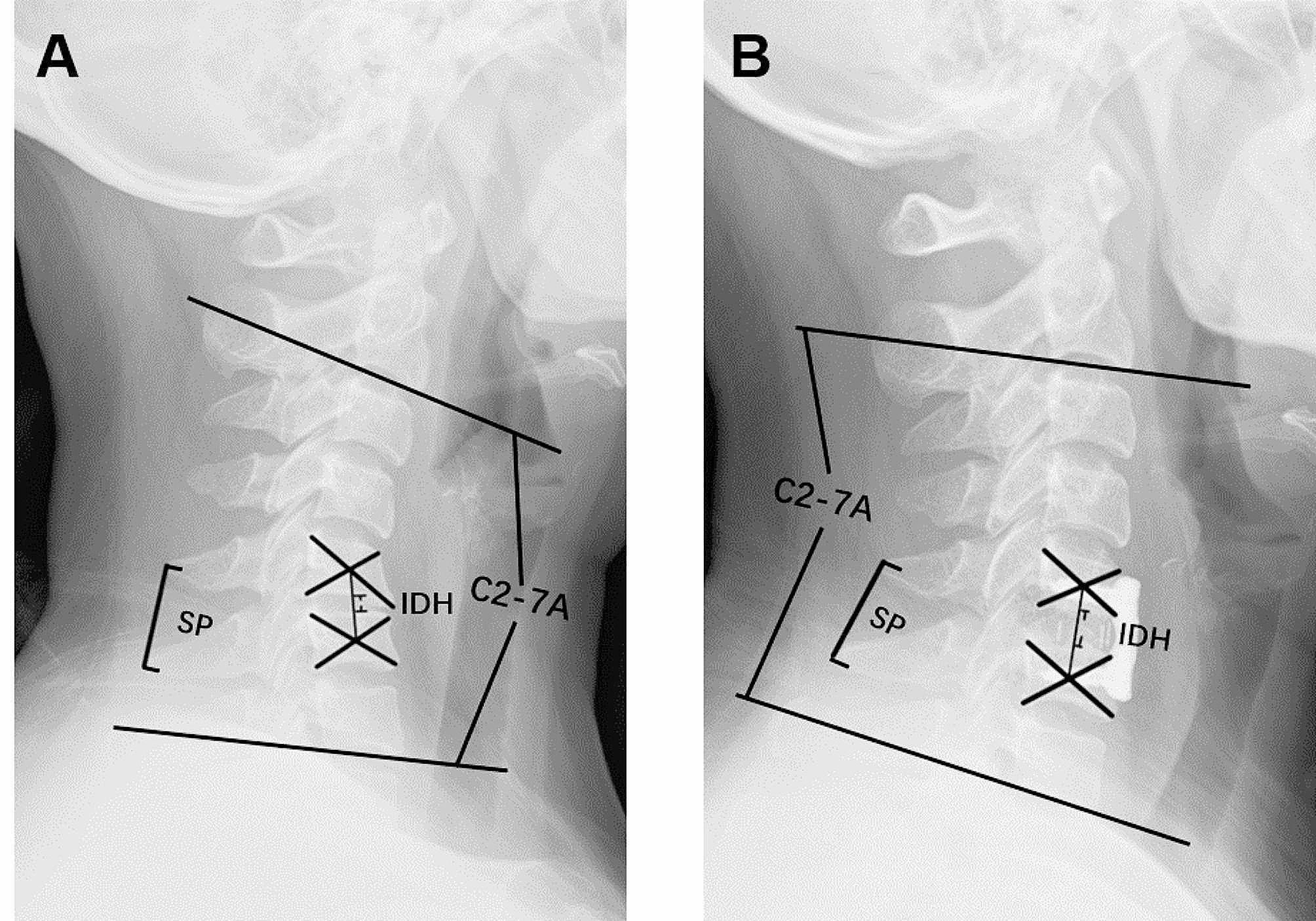
The definition of radiographic parameters on lateral radiographs, preoperatively (**A**) and postoperatively (**B**). C2-7 A was defined as the angle between the sacral endplates of C2 and C7; IDH was defined as IDH = the distance between the center of the upper and lower vertebrae - the distance between the center of the upper vertebral body and its lower endplate - the distance between the center of the lower vertebral body and its upper endplate. SP was the distance between the most posterior and caudad point of one spinous process to the same point on the adjacent spinous process

All plain lateral radiographs were taken by trained radiologists. Patients were required to stand erect and look ahead. Additionally, patients were instructed to keep their shoulders as low as possible so that the C7 vertebral body could be included in the radiographs [8]. Radiographic parameters included IDH, SP of the operated segment, and C2-7 A on lateral radiographs (Fig. 1). IDH was defined as the distance between the center of the upper and lower vertebrae - the distance between the center of the upper vertebral body and its lower endplate - the distance between the center of the lower vertebral body and its upper endplate. SP was the distance between the most posterior and caudad point of one spinous process to the same point on the adjacent spinous process. C2-7 A was defined as the angle between the sacral endplates of C2 and C7. For C2-7 A, kyphosis is considered a positive angle, while lordosis is considered a negative angle. The change in IDH, SP, and C2-7 A were defined as follows: change in IDH = postop IDH – preop IDH, change in SP = postop SP – preop SP, change in C2-7 A = postop C2-7 A – preop C2-7 A.

### Data analysis

Descriptive statistics involving normally distributed, continuous measures were expressed as the mean ± standard deviation (SD). Differences in baseline characteristics were tested using the two-tailed t-test for continuous variables, the Mann-Whitney U test for non-normally distributed variables, and the χ2 test for categorical variables. Spearman correlation analysis was also used to investigate the association of dysphagia with gender, age, BMI, operation time, blood loss, change in IDH, change in C2-7 A, and change in SP.

Receiver-operating characteristic (ROC) curves for change in IDH and change in C2-7 A were generated, and the areas under the curve (AUC) were calculated to identify and compare the accuracy of these two parameters for predicting postoperative dysphagia after single-level ACDF. By the way, a parameter was determined to have high accuracy when its AUC was more than 0.9, whereas 0.7 to 0.9 indicates moderate accuracy, 0.5 to 0.7 indicates low accuracy, and 0.5 a chance result [14, 15]. The AUC of these two ROC curves was compared using the method described by DeLong et al. [16]. All statistical analyses were performed using SPSS, version 22.0.

## Results

**Table 1:** Demographic and descriptive data (*n* = 217)

Descriptive Statistics	Mean	SD	Minimum	Maximum
Age (year)	56.23	8.79	36	77
BMI (Kg/m^2^)	24.35	3.50	18.90	38.52
Operation time (min)	87.24	15.74	60	135
Blood loss (ml)	34.10	15.57	5	100
Preop C2–7 A (degree)	11.18	6.74	-4.2	34.9
Postop C2–7 A (degree)	16.00	7.12	3.9	44.8
Change of C2–7 A (degree)	4.82	4.95	-6.4	27.4
Preop IDH (mm)	5.47	1.09	2.7	8.2
Postop IDH (mm)	8.31	0.53	6.9	9.9
Change of IDH (mm)	2.84	0.92	0.5	5.3
Preop SP (mm)	19.20	5.37	6.9	30.6
Postop SP (mm)	17.66	5.28	6.5	30.7
Change of SP (mm)	-1.54	3.67	-13.3	6.3

BMI: Body Mass Index; Preop: Preoperative; Postop: Postoperative;

C2–7 A: the angle between the sacral endplates of C2 and C7; IDH = the distance between the center of the upper and lower vertebrae - the distance between the center of the upper vertebral body and its lower endplate - the distance between the center of the lower vertebral body and its upper endplate; SP: the distance between the most posterior and caudad point of one spinous process to the same point on the adjacent spinous process

**Fig. 2:**
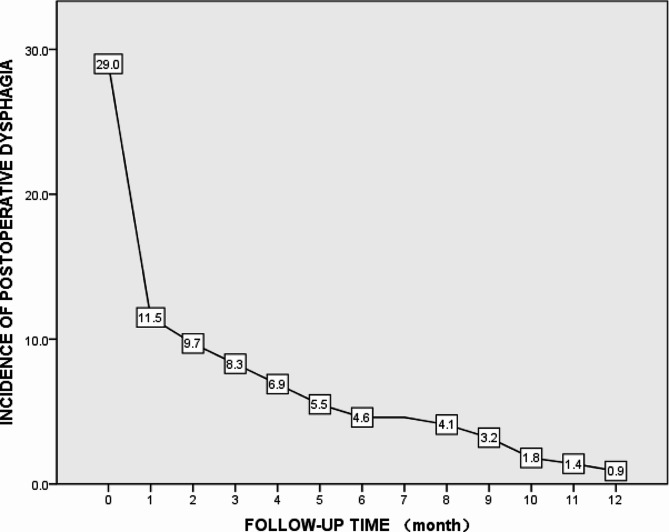
A line graph: the incidence of postoperative dysphagia after single-level ACDF over follow-up time (1 year)

Incidence and Severity of Postoperative Dysphagia after Single-Level ACDF:

The incidence of immediate postoperative dysphagia after single-level ACDF was 29.0% (63/217) and sharply decreased to 11.5% in the first month. Notably, only two patients (0.9%) experienced dysphagia persistently for more than a year (Fig. 2). Among the 63 patients who developed postoperative dysphagia, the incidence rates for “mild dysphagia,” “moderate dysphagia,” and “severe dysphagia” were 44.4% (28/63), 31.7% (20/63), and 23.8% (15/63), respectively, as per the dysphagia grading system defined by Bazaz et al. [17]

Univariate Analysis between Dysphagia and Non-dysphagia Patients:

In comparing dysphagia and non-dysphagia patients (Table 2), females (*P* = 0.016) were significantly associated with dysphagia, whereas age, BMI, operation time, and blood loss did not show significant associations with postoperative dysphagia (*P* > 0.05).

Change in C2-7 A, Change in IDH, and Change in SP:

Comparing dysphagia and non-dysphagia patients (Table 2), both change in C2-7 A and change in IDH (*P* = 0.000 and *P* = 0.001, respectively) exhibited significant differences. Furthermore, it was observed that when change in IDH was ≥ 3 mm and change in C2-7 A was ≥ 5 degrees, the likelihood of postoperative dysphagia was significantly greater than when change in IDH was < 3 mm (*P* = 0.000) and change in C2-7 A was < 5 degrees (*P* = 0.000). Surprisingly, no significant difference was found between the development of dysphagia and the change in SP (*P* > 0.05).

**Table 2:** Comparison between dysphagia and non-dysphagia patients (*n* = 217)

Parameters	None dysphagia (*n* = 154) (Mean ± SD)	Dysphagia (*n* = 63) (Mean ± SD)	Significance (2-tailed)
Age (year)	56.26 ± 9.12	56.16 ± 7.99	0.939
BMI (Kg/m^2^)	24.33 ± 3.43	24.41 ± 3.71	0.873
Operation time (min)	86.46 ± 16.10	89.08 ± 14.78	0.268
Blood loss (ml)	33.15 ± 15.99	36.43 ± 14.35	0.158
Preop C2–7 A (degree)	11.61 ± 6.44	10.16 ± 7.37	0.149
Postop C2–7 A (degree)	15.54 ± 6.25	17.14 ± 8.86	0.131
Change of C2–7 A(degree)	3.93 ± 3.69	6.99 ± 6.72	**0.000**
Preop IDH (mm)	5.56 ± 0.89	5.25 ± 1.45	0.057
Postop IDH (mm)	8.27 ± 0.48	8.41 ± 0.64	0.073
Change of IDH (mm)	2.71 ± 0.81	3.16 ± 1.09	**0.001**
Preop SP (mm)	19.28 ± 5.74	18.99 ± 4.36	0.718
Postop SP (mm)	17.72 ± 5.29	17.52 ± 5.29	0.800
Change of SP (mm)	−1.56 ± 3.71	−1.47 ± 3.59	0.870
(%) Female	34.6	19.4	**0.016**
(%) Male	36.4	9.7	
(%) Change of IDH ≥ 3 mm	20.7	20.3	**0.000**
(%) Change of IDH < 3 mm	50.2	8.8	
(%) Change of C2-7 A ≥ 5°	22.6	22.1	**0.000**
(%) Change of C2-7 A < 5°	48.4	6.9	

Spearman correlation analysis between the incidence of dysphagia gender, age, BMI, operation time, blood loss, change in C2-7 A, change in IDH, and change in SP(Table 3).

**Table 3:** Spearman correlation analysis

Risk Factors	correlation coefficients	Sig.
Gender	0.169	**0.012**
Age	−0.033	0.629
BMI	−0.006	0.934
Operation time	0.091	0.180
Blood loss	0.111	0.102
Change of C2–7 A	0.322	**0.000**
Change of IDH	0.238	**0.000**
Change of SP	0.005	0.939

Spearman correlation analysis revealed that the increased likelihood of postoperative dysphagia persisted with increased change in IDH (*P* = 0.000), change in C2-7 A (*P* = 0.000), and female gender (*P* = 0.012).

Comparison of Predictive Accuracy of Postoperative Dysphagia: Change in IDH and Change in C2-7 A (Table 4).

**Table 4:** The areas under the ROC curve (AUC)

Parameter	AUC	SE	Sig.	95% CI
Change of C2–7 A	0.705	0.045	**0.000**	(0.618, 0.792)
Change of IDH	0.651	0.045	**0.000**	(0.562, 0.740)
Change of C2–7 ≥ 5°	0.722	0.038	**0.000**	(0.647, 0.796)
Change of IDH ≥ 3 mm	0.703	0.040	**0.000**	(0.625, 0.781)

ROC: Receiver-operating characteristic; SE: standard error;

CI: confidence interval; The progressive significance of these four parameters, change of C2–7 A, change of IDH, change of C2–7 A ≥ 5°, change of IDH ≥ 3 mm were less than 0.001 compared with 0.5

**Fig. 3:**
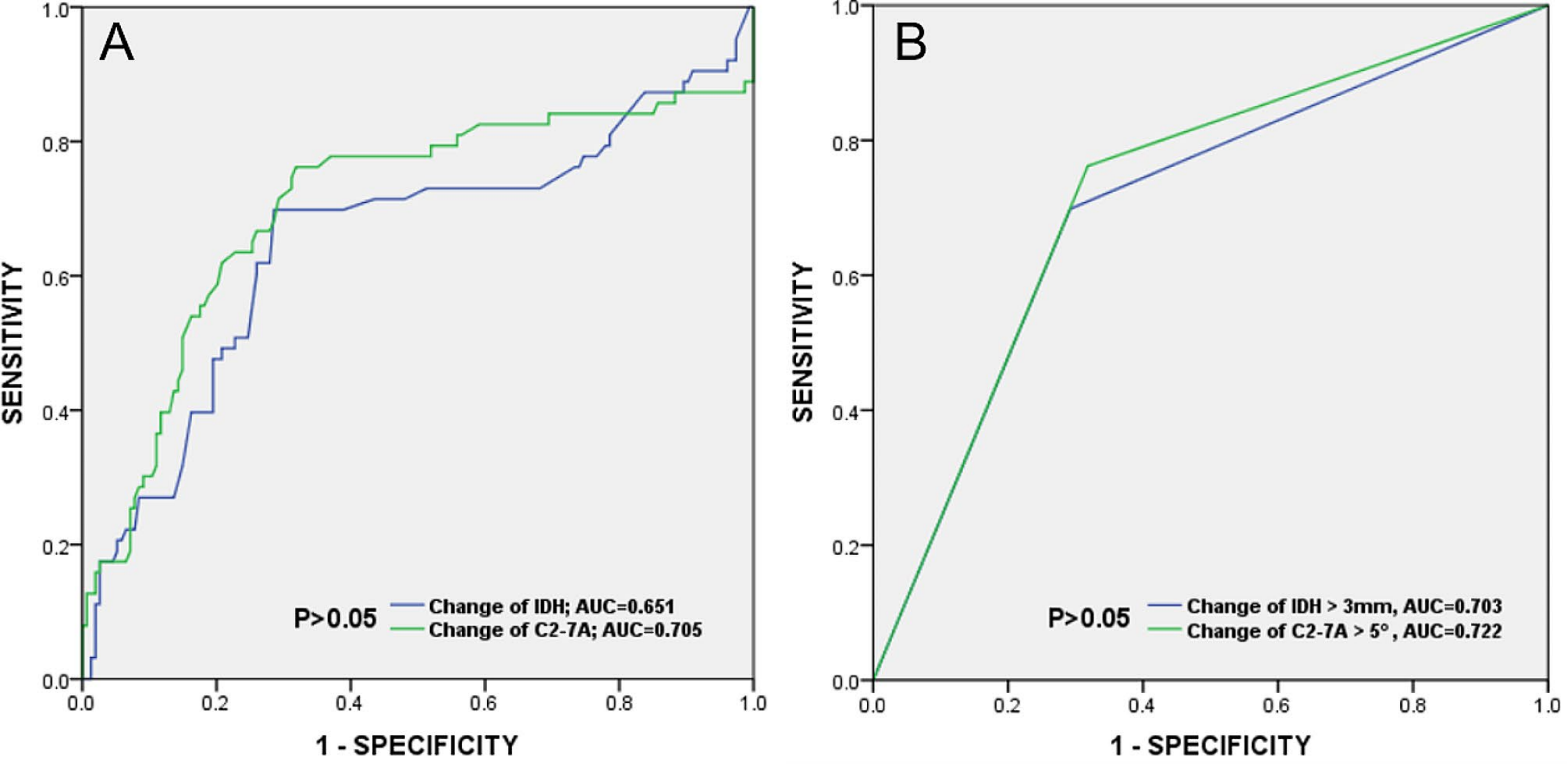
(**A**)Receiver-operating characteristic (ROC) curves for change of IDH and change of C2-7 A; (**B**) ROC curves for change of IDH ≥ 3 mm and change of C2-7 A ≥ 5 degrees. The areas under the curve (AUC) for change of IDH, change of C2-7 A, change of IDH ≥ 3 mm and change of C2-7 A ≥ 5 degrees were 0.651, 0.705, 0.703 and 0.722, respectively. No significant difference was observed between the AUC value of IDH change and C2-7 A change, IDH change ≥ 3 mm and C2-7 A change ≥ 5 degrees, respectively by DeLong test (*P* > 0.05)

Based on the analysis of ROC curves (Fig. 3) and AUC values (Table 4), change in IDH, change in C2-7 A, change in IDH ≥ 3 mm, and change in C2-7 A ≥ 5 degrees demonstrated low-to-moderate accuracy as predictors of postoperative dysphagia after single-level ACDF. The significance of these four parameters was less than 0.001 (Table 4), suggesting their reliability as predictive landmarks for postoperative dysphagia. Change in C2-7 A (AUC = 0.705) exhibited slightly higher predictive accuracy than change in IDH (AUC = 0.651), although no significant difference was found between the AUC of these two parameters. Similarly, change in C2-7 A ≥ 5 degrees (AUC = 0.722) showed slightly higher predictive accuracy than change in IDH ≥ 3 mm (AUC = 0.703), with no significant difference observed between the AUCs of C2-7 A change and IDH change (Fig. 3A), and C2-7 A change ≥ 5 degrees and IDH change ≥ 3 mm (Fig. 3B).

## Discussion

To our knowledge, this is the first study that specifically clarifies the relationship between the change of IDH and postoperative dysphagia after single-level ACDF with cervical lateral radiographs. Importantly, among several independent variables studied in our research, we found that females, change of IDH, and change of C2-7 A had an independent influence on postoperative dysphagia. In addition, age, BMI, operation time, and blood loss were not independently associated with postoperative dysphagia.

It is well known that the goals of ACDF are to restore the middle-lower curvature and height. It has been published that the change of C2-7 A is a predictive factor for postoperative dysphagia after ACDF [7–9], and when the change of C2-7 A is greater than 5°, the chance of developing postoperative dysphagia is significantly higher [8, 9]. Besides, no relationship was found between the change of C2-7 A and the degree of dysphagia [8, 9]. The reason why patients who had a larger change of C2–7 A had a greater chance of developing postoperative dysphagia is that the overenlargement of cervical lordosis may cause posterior pharyngeal wall bulging, reducing pharyngeal space and impairing pharyngeal squeeze and laryngeal elevation [8, 18]. However, it suggested that a new and simple landmark was needed to facilitate the assessment of cervical alignment for orthopedists [19], and the ideal cervical alignment could not be predicted by the change of C2-7 A alone [14] because it can only reflect changes in cervical curvature rather than changes in cervical height due to ACDF.

To solve this problem, IDH and SP were included in our study. There was no doubt that the dilation of the intervertebral space and the implantation of the interbody fusion cage lead to intervertebral height enlargement. However, the relationship between postoperative dysphagia and changes in the above parameters has not been studied. Comparing the first postoperative and preoperative plain lateral radiographs, our results of the average changes of IDH and SP are generally in good agreement with previous data. For example, it is reported that the mean increase in IDH was 2.62 mm [11], and the mean change in SP was − 0.8 mm [20].

In this study, as expected, we found that the incidence of dysphagia was significantly and independently associated with a higher change of IDH and change of C2-7 A after adjustment for various factors. Besides, when the change of IDH is ≥ 3 mm and the change of C2-7 A is ≥ 5 degrees, the incidence of postoperative dysphagia after single-level ACDF significantly increased. To date, very few radiological studies have directly investigated the independent influence of IDH and SP on postoperative dysphagia. It has been previously reported that IDH loss causes ventral angulation and eventual loss of cervical lordosis [13] during cervical degeneration, and the total disc height loss score was negatively associated with C2–C7 lordosis in a large-scale radiologic study of 865 [10]. On the contrary, C2–C7 lordosis was also restored when central interbody fusion was conducted to enlarge IDH during ACDF. That might be the reason why surgeons prefer to enlarge the IDH as much as possible. As a result, both C2–7 lordosis and IDH were restored perfectly. In a previous study, it was published that the change of C2-7 A was a significant predictive factor for postoperative dysphagia after ACDF because the overenlargement of cervical lordosis may cause posterior pharyngeal wall bulging, reducing pharyngeal space and impairing pharyngeal squeeze and laryngeal elevation [8, 18], which might be the results caused by the overenlargement of C2–7 and IDH cooperatively.

Our results showed that no relationship was found between the change of SP and the development of dysphagia. Yet, no study has directly explored the role of SP in postoperative dysphagia after ACDF. Commonsense reasoning suggests that the change of SP increases with increased IDH and decreased C2-7 A. Similarly, C2–C7 length (C2-7 L), a parameter increased with increased IDH and decreased C2-7 A, was defined as C2-C7 posterior vertebral body length [7] and reported that the mean change in C2–C7L was 1.7 mm after ACDF, but there was no significant difference between the change of C2-7 L and dysphagia after ACDF.

In our data, we observed that females could be considered a risk factor for postoperative dysphagia. Currently, the sex-related difference in the incidence of postoperative dysphagia after ACDF also remains controversial. For example, it was suggested that females were significantly more likely to experience postoperative dysphagia than males [21]. On the contrary, some studies showed no relationship between gender and postoperative dysphagia [7–9, 22]. In our opinion, the anatomical differences, variations in sample size, and inclusive criteria of patients might contribute to this controversy.

This study has several limitations. First, theoretically, multicenter studies are more conclusive compared to our unicentral study. Second, our study only analyzed the sagittal parameters of preoperative and the first postoperative lateral radiographs. Generally speaking, the results would be more conclusive if more lateral radiographs postoperatively at different follow-up times were involved in our study. Third, the first postoperative radiographs of patients were not taken immediately after ACDF because the drainage tube had to be removed before taking the radiographs. Fourth, our study only involved single-level ACDF, preventing us from discussing the impact of the number of ACDF levels. Fifth, we did not take the effect of ethnicity on the sagittal alignments of the cervical spine into consideration, making our results potentially not globally generalizable [23]. Lastly, our study did not include lateral radiographs of the full spine, and the relationship between postoperative dysphagia and other global sagittal parameters, such as thoracolumbar alignments, could not be discussed [10].

## Conclusion

The change in intervertebral disc height (IDH) could be considered a risk factor for postoperative dysphagia after single-level ACDF. A change in IDH ≥ 3 mm should be avoided to ensure a certain surgical effect.

## Data Availability

Datasets are available through the corresponding author upon reasonable request.

## Abbreviations

IDH: Intervertebral Disc Height
ACDF: Anterior Cervical Discectomy and Fusion
SP: spinous process distance
C2-7A: C2-7 angle
NIS: National Inpatient Sample
SD: standard deviation
ROC: Receiver-operating characteristic
AUC: areas under the curve
C2-7L: C2–C7 length
